# Effect of Three-Dimensional Printed Personalized Moisture Chamber Spectacles on the Periocular Humidity

**DOI:** 10.1155/2016/5039181

**Published:** 2016-10-24

**Authors:** Chan Hee Moon, Jae Yong Kim, Myoung Joon Kim, Hungwon Tchah, Byeong Gak Lim, Jin Kwon Chung

**Affiliations:** ^1^Department of Ophthalmology, Asan Medical Center, University of Ulsan College of Medicine, Seoul, Republic of Korea; ^2^Department of Ophthalmology, Aerospace Medical Center, Republic of Korea Air Force, Cheongju, Republic of Korea; ^3^Goodeye Clinic, Incheon, Republic of Korea; ^4^Department of Ophthalmology, Soonchunhyang University College of Medicine, Soonchunhyang University Hospital, Seoul, Republic of Korea

## Abstract

*Purpose.* To assess the effect of three-dimensional (3D) printed personalized moisture chamber spectacles (PMCS) on the periocular humidity.* Methods.* Facial computed tomography (CT) scanning was conducted on 10 normal subjects. PMCS was designed based on volume rendered CT images and produced using a 3D printer. Periocular humidity of PMCS and commercially available uniformed moisture chamber spectacles (UMCS) were measured for 30 minutes via microhydrometer.* Results.* The mean ambient humidity was 15.76 ± 1.18%. The mean periocular humidity was 52.14 ± 3.00% in PMCS and 37.67 ± 8.97% in UMCS. The difference was significant (*P* < 0.001). Additionally, PMCS always demonstrated lower humidity than dew points.* Conclusion.* PMCS made by 3D printer provides appropriate fitness for the semiclosed humid chamber. PMCS showed higher performance than UMCS. The wearing of PMCS would be an effective method to provide high enough periocular humidity in low humidity environment.

## 1. Introduction

Dry eye is a common ocular disease prompting millions of individuals to seek ophthalmological care [1]. Artificial tear supplements are the most common form of treatment for dry eye. Moisture chamber spectacles are not used habitually in the treatment of dry eye; however they can be considered as an adjunct therapy in the case of severe dry eye when patients are recalcitrant to other means of treatment or special environment in which frequent instillation of eyedrops is unapplicable.

Korb et al. reported that increased periocular humidity using modified swim goggles resulted in an increase in tear film lipid layer thickness and moderate to total relief of dry eye symptoms [2]. Tsubota et al. attached small moistened sponges to side panels of spectacles to increase the moisture level near the cornea. Moisture chamber spectacles showed 12.9% increase of periocular humidity and all enrolled dry eye patients noticed symptomatic relief with significantly decreased use of artificial tear [3].

Unlike general spectacles, moisture chamber spectacles need to be personalized in shape and size of frame for best performance. The tear retention effectiveness of moisture chamber spectacles is determined by the extent of proper semiclosed chamber formation. The formation of an appropriate chamber depends on how well the spectacles fit the facial contour. There are several commercially available moisture chamber spectacles; however they are single size or minimally adjustable. Previous studies were also performed using spectacles with single or several size variations [2, 3].

Three-dimensional (3D) printing was invented in the 1980s and has shown marked growth in recent years. 3D printing has been advancing in customized product printing and has proved to be effective for medical applications [4–6]. Recently, 3D printing also has been highlighted in the field of ophthalmology [7–9]. One of the biggest advantages of 3D printing is to facilitate the production of personalized goods at affordable prices that are comparable with traditionally manufactured items.

In this study, personalized moisture chamber spectacles (PMCS) were produced using facial computed tomography (CT) scanning and 3D printing technology. The effect of PMCS on periocular humidity was evaluated and the clinical implication was discussed.

## 2. Methods

### 2.1. Subjects

This study was approved by the Institutional Review Board of research ethical committees of Aeromedical Center, Republic of Korea Air Force, and conformed to the tenets of the Declaration of Helsinki (ASMC-15-IRB-001). Ten normal subjects who have no history of evidence of ophthalmic disease were recruited.

### 2.2. Facial CT Scanning

**Figure 1:**
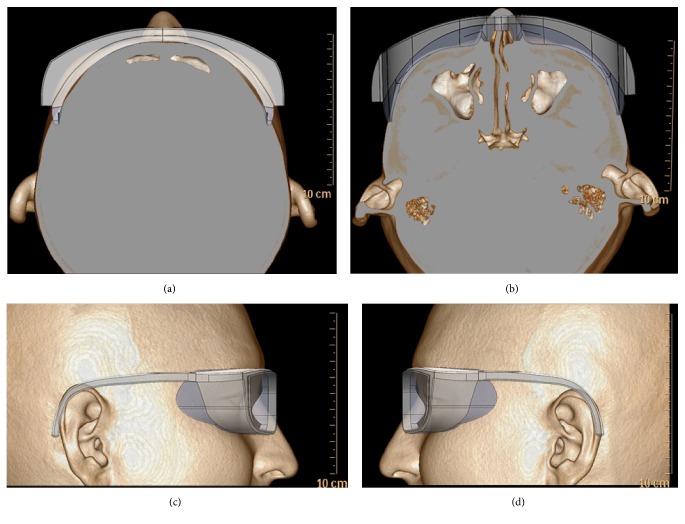
Personalization of moisture chamber spectacles using CT scan images. (a) Top, (b) bottom, (c) right side, and (d) left side.

Facial CT scanning for PMCS was conducted on the participants. Facial CT scanning was performed using a 64-channel CT scanner (Philips Brilliance 64; Philips Healthcare, Best, Netherlands). After conventional CT scan, 3D reconstruction was processed and facial contour was obtained using volume rendering tool, which was incorporated in the workstation (Figure 1).

### 2.3. Production of the PMCS

**Figure 2:**
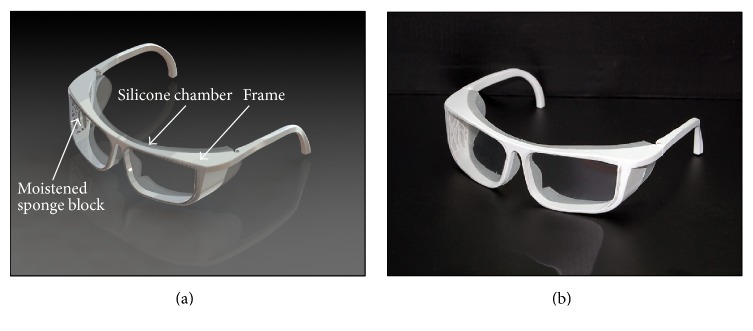
Rendering images and 3D printout of personalized moisture chamber spectacles. (a) Rendering images on CAD program. (b) 3D printed-out product.

The spectacles were designed using computer-aided design (CAD) program (SolidWorks 2013; SolidWorks Corp., MA, USA). PMCS consisted of mainly 4 parts, that is, frame, side panel, moistened sponge block, and silicone chamber (Figure 2). All parts are personalized in size and shape, based on CT images. Frame and side panel were produced by 3D printer using polylactic acid material (Robox; CEL technology, North Somerset, UK). The width of the frame was set in bitemporal distance. The length of the temple was set in straight distance between frame and helix of ear for a little tight fitting. Posterior surface of silicone chamber was designed identically to the face contour. The side panels were designed to contain room for moistened sponge block. The inner side panel was made as many geographically perforated structures for providing humidity through passive evaporation from the moistened sponge. High absorptive melamine foam which consists of a formaldehyde-melamine-sodium bisulfite copolymer was used for moisture sponge block (Magic Eraser; The Procter & Gamble Company, Ohio, USA). Sponge block had an average surface area of 778.5 ± 13.6 mm^2^ and held 0.85 g of distilled water. Silicone part of moisture chamber was constructed on CAD and the mold was printed. Then silicone (Mold Master Ultra; Molkang, Paju, Korea) was infused into the mold and silicone chamber was formed.

### 2.4. Evaluation of Periocular Humidity and Temperature

**Figure 3:**
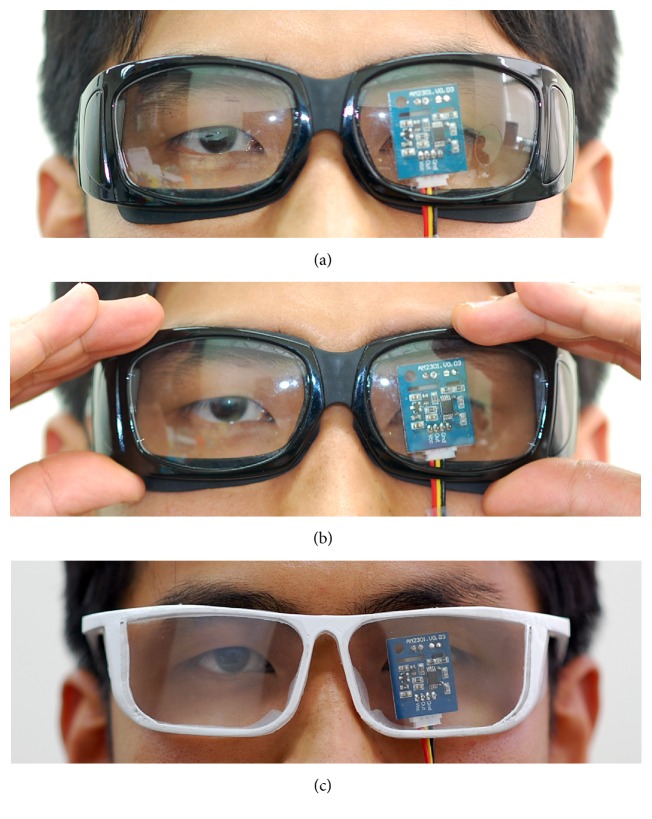
Wearing of moisture chamber spectacles. (a) Natural wearing of uniformed moisture chamber spectacles. (b) Pressurized wearing of uniformed moisture chamber spectacles. (c) Natural wearing of personalized moisture chamber spectacles.

The present study was designed as cross-over test. The subjects wore a sequence of different moisture chamber spectacles. Commercially available uniformed moisture chamber spectacles (UMCS) (Eyeeco, CA, USA) and PMCS were worn naturally for 30 minutes while periocular humidity and temperature were measured. UMCS were worn once again with gentle pressure on the spectacles frame in order to measure periocular humidity with nearly perfect sealing of moisture chamber. The order of the wearing spectacles was randomized. There was an interval of 30 minutes, prior to wearing other spectacles. The microhydrothermometer (AM2301; Aosong Electronics Co., Guangzhou, China) was attached to the backside of the spectacles lens (Figure 3). Left eyes were used for the analysis. The other identical hydrothermometer was used for ambient measurements. The humidity and temperature were measured every 30 seconds for 30 minutes. Room temperature and humidity were controlled at 22-23°C and 15-16% with thermoregulator and dehumidifier.

### 2.5. Calculation of Dew Points

The excessively high periocular humidity induces condensation on the spectacles lens and visual blur. The dew point is the temperature at which dew forms. A simple equation for the relationship between relative humidity and the dew point is as follows [10] (*t*_*d*_ = dew point, *t* = temperature, and RH = relative humidity):

(1) $$\begin{array}{c} t_{d}=t-\left(\;\frac{100-RH}{5}\;\right) \end{array}$$

or

(2) $$\begin{array}{c} RH=100-5\left(\;t-t_{d}\;\right). \end{array}$$

The periocular humidity level which starts to cause condensation on the spectacles can be calculated by substituting periocular temperature for “*t*” and ambient temperature for “*t*_*d*_.” The calculated RH and measured periocular humidity were compared.

### 2.6. Statistical Analysis

A repeated measures analysis of variance (rANOVA) and Bonferroni post hoc test were performed for comparisons among measurements. Shapiro-Wilk was used for normality test. Statistical analysis was conducted, using SPSS Statistics Version 21 (IBM Corporation, Somers, NY). All tests were two-tailed and *P* < 0.05 was considered statistically significant.

## 3. Results

The mean age of participants was ±years. Eight subjects were males and 2 were females. Facial characteristics including interpupillary distance, bitemporal distance, and temple length were described in Table 1.

### 3.1. Humidity

**Figure 4:**
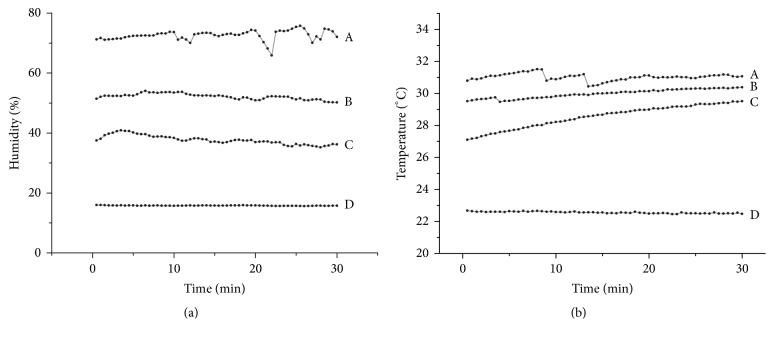
Measurements of periocular humidity and temperature over time. The average of periocular humidity and temperature over time in 10 volunteers. (a) Pressurized wearing of uniformed moisture chamber spectacles, (b) natural wearing of personalized moisture chamber spectacles, (c) natural wearing of uniformed moisture chamber spectacles, and (d) ambient humidity.

**Figure 5:**
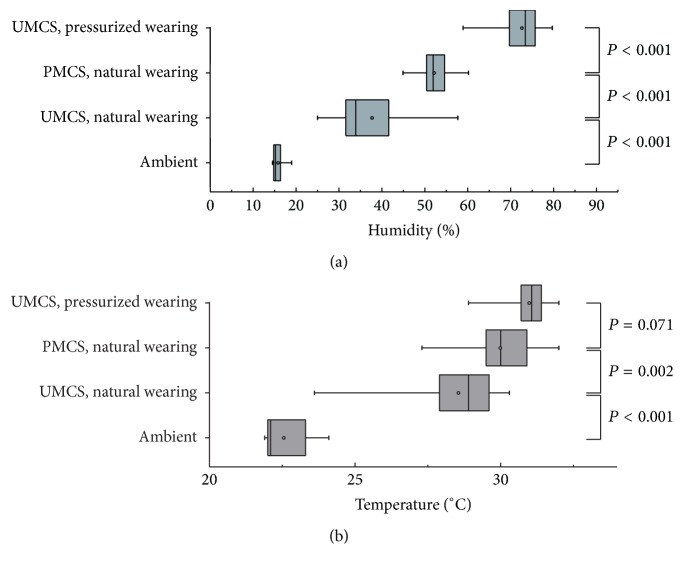
Comparisons of periocular humidity and temperature with various spectacles wearing. Box plots show minimum and maximum (whiskers), 25% and 75% quartiles (box), the median (line in the box), and mean (dot in the box).

The hydrometer had an accuracy of ±3% RH and a repeatability of ±1% RH. Measurements of periocular humidity over time were shown in Figure 4(a). The mean ambient humidity was 15.76 ± 1.18%. The mean periocular humidity was highest with pressurized wearing of UMCS (72.58 ± 4.42%), followed by natural wearing of PMCS (52.15 ± 3.00%) and natural wearing of UMCS (37.67 ± 8.96%). The differences of humidity measurements with various spectacles were significant (*P* < 0.001), (Figure 5(a)).

### 3.2. Temperature

The thermometer had an accuracy of ±0.5°C and a repeatability of ±0.3°C. Measurements of periocular temperature over time were shown in Figure 4(b). The average ambient temperature was 22.55 ± 0.76°C. The mean periocular temperature was highest with pressurized wearing of UMCS (30.98 ± 0.63°C), followed by natural wearing of PMCS (29.98 ± 1.21°C) and natural wearing of UMCS (28.55 ± 1.38°C). Periocular temperature with natural wearing of PMCS was significantly higher than natural wearing of UMCS (*P* = 0.002). However, the difference of periocular temperature between pressurized wearing of UMCS and natural wearing of PMCS was not significant (*P* = 0.071), (Figure 5(b)).

### 3.3. Condensation

**Figure 6:**
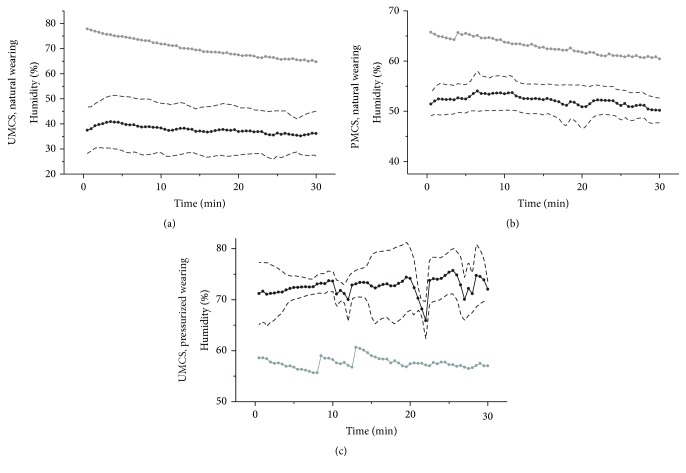
Measured periocular humidity and calculated condensation humidity. Measured periocular humidity (black line), ±1 standard deviation of humidity measurements (dash line), and calculated condensation humidity (gray line).

Measured periocular humidity and calculated humidity level that starts to cause the condensation on the spectacles were shown in Figure 6. When periocular humidity becomes greater than calculated condensation humidity, it is indicative of condensation. In this study, measured periocular humidity of natural wearing of UMCS and PMCS demonstrated lower value than calculated condensation humidity at all time points; furthermore, no participants complained of blurred vision due to condensation in practice. However, measured periocular humidity of pressurized wearing of UMCS showed higher value than calculated condensation humidity at all time, and all subjects experienced visual blur.

## 4. Discussion

The wearing of moisture chamber spectacles can be helpful to improve ocular discomfort associated with dry eye. Based on the definition from the dry eye workshop (DEWS), dry eye disease is defined as multifactorial disease that is accompanied by increased osmolarity of the tear film and inflammation of the ocular surface [11]. The DEWS established increased tear osmolarity and tear film instability, followed by goblet cell, glycocalyx mucin loss, and epithelial damage as core mechanisms of dry eye. High evaporation is one of the major causes of tear hyperosmolarity [11]. Tear evaporation rate depends on mass transfer through both the coating lipid layer and ambient air [12]. McCulley et al. reported that 10% decrease in relative humidity resulted in 28.33% to 59.42% increase in tear evaporation [13]. Uchiyama et al. showed that a decrease of relative humidity from 40–45% to 20–25% resulted in 99.72% increase in tear evaporation rate [14]. Additionally, short tear breakup time dry eye is known to be a major part of visual display terminals- (VDT-) associated dry eye [15]. It developed in 66% of VDT workers and has become increasingly common [16]. Environmental factors such as air drafts and low humidity in the air are known to progressively increase tear water evaporation and promote faster thinning of the precorneal tear film and the consequent ocular surface damage [17].

In this study, PMCS increased absolute value of 36.39% periocular humidity, as compared to the ambient humidity. Moisture chamber spectacles may be particularly helpful to reduce artificial tear use for severe dry eye, involving Sjögren's syndrome or lagophthalmos patients, and ultra-low humidity environmental worker including laboratory employee [18] or aircraft cabin crew [19].

Appropriate fit is the most critical aspect in moisture chamber spectacles. The loose fitness of spectacles reduces relative humidity in the moisture chamber. In the present study, PMCS showed significantly higher periocular humidity than UMCS, as predicted. Additionally, UMCS demonstrated wide individual differences of periocular humidity (standard deviation = 8.96), while PMCS showed narrow individual variations (standard deviation = 3.00). In contrast, tight fitness of spectacles induces excessively high relative humidity, resulting in condensation as well as discomfort. Moisture chamber should be a semiclosed, and not a completely closed space due to condensation. In this study, pressurized wearing of UMCS which formed near completely closed space showed higher mean periocular humidity than condensation humidity. Though the higher periocular humidity provides greater tear retention effect, the spectacles inducing condensation without antifog material are not practical for daily wear. PMCS provided sufficiently high periocular humidity; however the measurements were always slightly lower than the condensation point. These results suggested that personalization of moisture chamber provides appropriate fit.

There were some limitations to this study. The present study had small sample size and did not include patient group and clinical evaluation. Further investigation will be needed to determine the clinical effectiveness of PMCS, involving subjective symptoms and objective ocular surface evaluation in patients with dry eye disease with larger sample size.

## 5. Conclusions

PMCS made by 3D printer provides appropriate fitness to form semiclosed humid chamber. PMCS increased absolute value of 36.39% periocular humidity without condensation as compared to the ambient humidity and demonstrated significantly higher performance than UMCS. The wearing of PMCS would be an effective method to provide highly enough periocular humidity in low humidity environment.

## Figures and Tables

**Table 1:** Demographic characteristics.

Number of participants	10
Age (year)	32 ± 10.1
Sex (male/female)	8/2
Interpupillary distance (mm)	62.4 ± 5.5
Bitemporal distance (mm)	149.3 ± 10.7
Temple length (mm)	81.5 ± 8.0
